# Remote ischemic conditioning in ST-elevation myocardial infarction as adjuvant to primary angioplasty (RIC-STEMI): study protocol for a randomized controlled trial

**DOI:** 10.1186/s13063-015-0937-1

**Published:** 2015-09-08

**Authors:** António Gaspar, Miguel Álvares Pereira, Pedro Azevedo, André Lourenço, Jorge Marques, Adelino Leite-Moreira

**Affiliations:** Department of Physiology and Cardiothoracic Surgery, Cardiovascular R&D Unit, Faculty of Medicine, University of Porto, Alameda Prof. Hernâni Monteiro, 4200-319 Porto, Portugal; Cardiology Department, Hospital of Braga, Braga, Portugal; Department of Cardiothoracic Surgery, Hospital of S. João, Porto, Portugal

## Abstract

**Background:**

ST-elevation myocardial infarction (STEMI) accounts for nearly one third of acute coronary syndromes. Despite improved STEMI patient care, mortality remains high, contributing significantly to the ischemic heart disease burden. This may partly be related to ischemia-reperfusion injury (IRI). Remote ischemic conditioning (RIC), through short cycles of ischemia-reperfusion applied to a limb, has been shown to reduce IRI in various clinical settings. Our primary hypothesis is that RIC will reduce adverse events related to STEMI when applied as adjunctive therapy to primary percutaneous coronary intervention (PCI).

**Methods/Design:**

“Remote ischemic conditioning in ST-elevation myocardial infarction as adjuvant to primary angioplasty” (RIC-STEMI) is an ongoing prospective, single-center, open-label, randomized controlled trial to assess whether RIC as an adjunctive therapy during primary PCI in patients presenting with STEMI can improve clinical outcomes. After enrollment, participants are randomized according to a computer-generated randomization schedule, in a ratio of 1:1 to RIC or no intervention, in blocks of four individuals. RIC is begun at least 10 min before the estimated time of the first balloon inflation and its duration is 30 min. Ischemia is induced by three cycles of inflation of a blood pressure cuff placed on the left lower limb to 200 mmHg and then deflation to 0 mmHg for another 5 min. Primary endpoint is a combined endpoint of death from cardiac cause or hospitalization for heart failure (HF) on follow-up (including device implantation: implantable cardioverter defibrillator, cardiac resynchronization and left ventricular assist device). Secondary endpoints are myocardial infarction (MI) size (estimated by the 48 h area under the curve of serum troponin I levels), development of Q-wave MI, left ventricular function (assessed by echocardiography within the first 3 days after admission), contrast-induced nephropathy, in-hospital mortality, all-cause mortality and, finally, major adverse cardiovascular events. Patients will have a minimum follow-up period of 12 months. From 11 March 2013 to 31 December 2014, 324 patients have been enrolled and randomized. We expect to complete enrollment of the 494 patients deemed necessary within 3 years.

**Trial registration:**

ClinicalTrials.gov identifier: NCT02313961; registered on 8 December 2014.

**Electronic supplementary material:**

The online version of this article (doi:10.1186/s13063-015-0937-1) contains supplementary material, which is available to authorized users.

## Background

Ischemic heart disease is the leading cause of mortality worldwide, accounting for over 7 million deaths per year [1]. ST-elevation myocardial infarction (STEMI) accounts for nearly one third of acute coronary syndromes. The widespread use of timely reperfusion, particularly with primary percutaneous coronary intervention, has resulted in a significant reduction in the acute STEMI mortality, reaching approximately 5 % in recent years [2]. However, this reduction of the in-hospital mortality also led to an increase in the incidence of chronic heart failure (HF) because many patients with severely depressed left ventricular function, who would have died in the acute STEMI phase in the past, now survive at the cost of severe HF [2]. As a result, 6-month mortality associated with STEMI remains unacceptably high, approximately 12 %, with an even higher mortality rate in higher risk patients [3, 4]. Consequently, attention is no longer solely aimed at reducing the acute mortality associated with STEMI but also to limit the downstream consequence of postinfarction HF [5]. Ischemia-reperfusion injury (IRI) is believed to account for up to 40-50 % of infarct size and may be a target to prevent evolution to heart failure after STEMI [6, 7]. Several pharmacological alternatives have been attempted to prevent IRI in promising animal experiments; nevertheless, clinical translation has been disappointing [8]. On the opposite side, ischemic conditioning (IC) by short cycles of ischemia-reperfusion applied before, during or after a major ischemic event has clearly been shown to attenuate IRI in various clinical scenarios. Moreover, even so-called remote IC (RIC), which are repeated bouts of limb ischemia, are cardioprotective, [9–11]. In 2010, Bøtker et al. showed improved myocardial salvage index as assessed by single photon emission computed tomography 30 days after PCI in patients randomly assigned to receive concomitant RIC [12], whereas Rentoukas *et al*. found higher proportions of ST-segment resolution with adjunctive RIC compared with PCI alone. However, significant reductions in troponin I peaks only reached statistical significance in a subgroup undergoing both RIC and morphine therapy combined with PCI [13]. More recently, Sloth et al. evaluated the long-term effect of RIC on the population initially analyzed by Bøtker et al. (166 patients underwent PCI with adjunctive RIC and 167 patients simply underwent PCI). The authors showed improved long-term prognosis for patients that underwent adjunctive RIC as regards the composite endpoint of adverse cardiac and cerebrovascular events: all-cause mortality, MI, readmission for HF, and ischaemic stroke/transient ischaemic attack [14]. However, although very promising, their results are inconclusive with regard to cardiovascular mortality and HF development, since the study was not powered to show differences in these clinical events. Large-scale studies addressing major adverse cardiovascular events are warranted [5, 7, 15].

The primary objective of our trial is to test the hypothesis that RIC will reduce adverse events related to STEMI, mainly by preventing evolution to post-infarction HF.

## Methods/Design

The trial protocol was developed in accordance with the SPIRIT (Standard Protocol Items: Recommendations for Interventional Trials) guidelines [16] (Appendix 1). Overview of the study design

RIC-STEMI is an ongoing prospective, single-center, open-label, randomized controlled trial to assess whether RIC as an adjunctive therapy during primary PCI in patients presenting with STEMI can improve clinical outcomes as assessed by a cardiac-caused death or hospitalization for heart failure (HF) for a minimum follow-up period of 12 months. According to IRI pathophysiology, RIC is expected to reduce infarct size and, consequently, post-infarction heart failure and the adverse events associated to it. In contrast, RIC is not expected to have any major effect on further ischemic and thrombotic events; therefore, other major cardiovascular events (MACE) such as new-onset MI, stroke and need for redo revascularization, either by coronary artery bypass grafting (CABG) or PCI, are not part of the primary endpoints.

### Setting and participants

All PCI eligible patients presenting with putative STEMI at our institution are screened for enrollment. Our hospital is a tertiary referral and academic center treating almost 300 STEMI patients per year.

Inclusion criteria are indicated as follows:

1.Patients ≥ 18 years old;
2.STEMI defined as chest pain (or epigastric pain) for more than 30 min and either (a) new ST elevation at the J point in two contiguous leads with the cut-off points of ≥0.2 mV in men or ≥0.15 mV in women in leads V2-V3 or ≥0.1 mV in all other leads or (b) new or presumed new left bundle branch block;
3.Symptom onset not more than 12 h before presentation; and
4.Willingness and capability to provide informed consent.

Exclusion criteria are indicated as follows:

1.Cardiogenic shock as defined by systemic hypotension (systolic arterial pressure (SAP) below 90 mmHg) and evidence of tissue hypoperfusion;
2.Postcardiac arrest status;
3.Need for mechanical ventilation;
4.Known peripheral artery disease or evidence of lower limb ischemia; or
5.Recent myocardial infarction (within 30 days).

Patients will also be excluded from further analysis if coronarography does not confirm STEMI or if CABG is required, according to the attending physician’s assessment.

### Randomization and interventions

After enrollment, participants are randomized according to a computer-generated randomization schedule, in a ratio of 1:1 to RIC or no intervention, in blocks of four individuals.

After the patient’s arrival in the catheterization laboratory (cath lab), a blood pressure cuff is placed on the left lower limb by one of the cath lab nurses. Because more than 95 % of our patients are catheterized using the radial artery, the left lower limb was chosen in order to not interfere with this approach. RIC is begun approximately 10 min before the estimated time of the first balloon inflation and its duration is 30 min. As reperfusion must be achieved as early as possible, RIC may be completed during and even after the angioplasty. Ischemia is induced by three cycles of inflation of the blood pressure cuff to 200 mmHg and then deflation to 0 mmHg for another 5 min (Appendix 2). Apart from temporary moderate pain in the treated thigh, RIC has been shown to be innocuous. All patients receive the standard of care therapy according to institutional guidelines, namely treatment with 250 mg aspirin intravenously, a P2Y_12_ inhibitor orally and 5000 IU unfractioned heparin intravenously before PCI. The choice of balloons, stent types and PCI procedure, as well as the use of glycoprotein IIb/IIIa inhibitors, is left to the discretion of the attending physician.

### Study endpoints

RIC is expected to reduce myocardial damage associated with IRI, preventing postinfarction heart failure.

The primary endpoint is a combined outcome of death from cardiac cause or hospitalization for HF on follow-up (including device implantation: implantable cardioverter defibrillator, cardiac resynchronization and left ventricular assist device). Secondary endpoints are myocardial infarction (MI) size (estimated by the 48 h area under the curve of serum troponin I levels), development of Q-wave MI, left ventricular function (assessed by echocardiography within the first 3 days after admission), contrast-induced nephropathy, in-hospital mortality, all-cause mortality and, finally, MACE on follow-up.

Patients will have a minimum follow-up of 12 months. Information will be obtained through medical record consultation and phone calls. Cardiac and noncardiac causes of death will be assigned by three consulting cardiologists who will not know which patients were submitted to RIC. Data will be reviewed by two independents researchers who also will not know which patients were submitted to RIC.

### Sample size

Sample size for the survival analysis was computed with the aid of IBM SPSS Sample-Power software with two-tailed type I error rate set at 0.05 and power at 0.80, conservatively admitting 5 % drop-outs during the RIC protocol and 5 % losses to follow-up. Considering an accrual period of 36 months, a minimum follow-up period of 12 months (maximum follow-up of 48 months), overall primary combined endpoint incidence of 14 % (data from our institution) and a treatment effect of 40 % (arbitrarily set as clinically relevant), we estimate we will need to enroll 224 patients per group (total of 448). Nevertheless, since 8 % of initially enrolled patients (data from our institution) will have unconfirmed STEMI, we will need to enroll 22 more patients per group (246) for a total of 492 patients.

### Data management and statistical analyses

Data will be recorded in an electronic database. Unique numeric identifiers will be assigned to all patients, and only this unique number will be included in the study database. Patients’ identification will be solely used for follow-up purposes. The investigators will ensure safekeeping of the patients’ data. Follow-up will be conducted through consultation of medical records and phone contact by trained investigators with large experience in completing participant follow-ups.

Primary and secondary endpoints will be analyzed according to intention-to-treat principles. Patient subgroups such as those presenting with a completely occluded artery, anterior STEMI and pain lasting longer than 3 hours will also be analyzed.

Statistical analyses will be performed using SPSS software. The chi-square test will be used to compare categorical variables, expressed as percentages. Continuous variables, expressed as means ± standard deviation, will be compared using the Student’s *t* test for those with a normal distribution, or the Mann-Whitney test for non-normally distributed data.

### Ethics

This study is being conducted in accordance with the Declaration of Helsinki 1964 as revised in 2013 and the International Conference of Harmonisation Guidelines for Good Clinical Practice.

Considering that STEMI is a medical emergency, little time is available. Eligible patients are orally informed and asked to participate in the study. Enrollment will be based on witnessed oral consent, and full written informed consent will be obtained by one of the investigators only after the acute phase has been addressed. Patients are notified at enrollment of their freedom to abandon the study at any time without consequences.

The study protocol (version HB-CARD-01; 20/11/2012) has been reviewed and approved by our Institution’s Ethical Committee (“Comissão de Ética para a Saúde do Hospital de Braga”), as verified with the board approval number 94/2012 (submitted and approved).

### Dissemination of results

The trial protocol and results will be published in peer-reviewed journals. The results of the trial will be reported in accordance with the CONSORT (Consolidated Standards of Reporting Trials) statement and its extension to non-pharmacological interventions [17, 18].

## Discussion

Despite its decline in recent years, STEMI mortality in European countries remains high, reaching at least 12 % at 6 months [3, 4]. Current mortality rates can hardly be further improved by PCI developments, strongly suggesting an important role of IRI, which can contribute to up to 40 to 50 % of the final infarct size and is, therefore, a main determinant of HF development [2, 5, 7, 15].

RIC is a safe noninvasive low cost intervention that has shown positive results in mitigating IRI in several clinical settings, including STEMI [12, 13]. So far, only one study evaluated the long-term prognosis of STEMI patients submitted to RIC [14]. However, this study was not initially powered to show an effect on hard clinical endpoints. As such, for the time being, although promising, RIC cannot be advised in evidence-based practice.

RIC-STEMI was designed to evaluate the impact of RIC as adjunctive to PCI in STEMI patients in the combined clinical outcome of cardiac-caused death or hospitalization for HF. Enrollment of the patients began in March 2013, and 324 patients were randomized in just under two years. Considering the number of patients usually referred to our institution, we expect to complete enrollment within 3 years.

## Trial status

RIC-STEMI is still recruiting participants.

From 11 March 2013 to 31 December 2014, 324 patients were enrolled and randomized for RIC-STEMI, but 29 of those were excluded from further analysis because STEMI was not confirmed on coronarography.

RIC was well tolerated by an overwhelming majority of patients.

## Additional files

Additional file 1: SPIRIT checklist (DOC 110 kb) Additional file 2: TIDieR checklist (DOCX 29 kb)

## Abbreviations

STEMI: ST-elevation myocardial infarction
IRI: ischemia-reperfusion injury
PCI: percutaneous coronary intervention
IC: ischemic conditioning
RIC: remote ischemic conditioning
HF: heart failure
MACE: major adverse cardiovascular events
CABG: coronary artery bypass grafting
MI: myocardial infarction
