# Supplementary material for: Remote ischemic conditioning in ST-elevation myocardial infarction as adjuvant to primary angioplasty (RIC-STEMI): study protocol for a randomized controlled trial
Source: Trials. 2015 Sep 8;16:398. doi: 10.1186/s13063-015-0937-1 (PMC4563839; doi:10.1186/s13063-015-0937-1)
Supplement: Additional file 1: SPIRIT checklist — (DOC 110 kb) [file 13063_2015_937_MOESM1_ESM.doc]

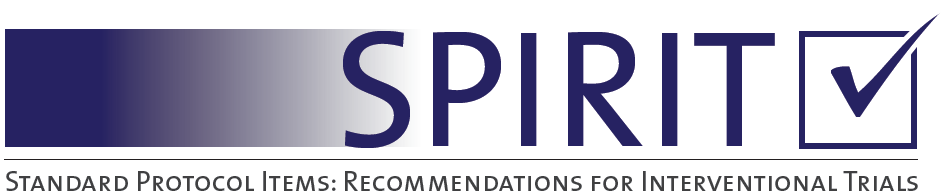


SPIRIT 2013 Checklist: Recommended items to address in a clinical trial protocol and related documents

| Section/item | | ItemNo | Description |
| --- | --- | --- | --- |
| **Administrative information** | | | |
| Title | 1 | | Remote ischemic conditioning in ST-elevation myocardial infarction as adjuvant to primary angioplasty (RIC-STEMI): study protocol for a randomized controlled trial (page 1, lines 2 and 3) |
| Trial registration | 2a | | Page 3; line 3: ClinicalTrials.gov NCT02313961 |
|  | 2b | | N/A |
| Protocol version | 3 | | Page 9, line 20: HB-CARD-01 (20/11/2012) |
| Funding | 4 | | Page 12, lines 2 -4 |
| Roles and responsibilities | 5a | | Page 11, line 18 |
|  | 5b | | Hospital de Braga; Lugar de Sete Fontes,  4710-243 São Victor, Braga |
|  | 5c | | N/A |
|  | 5d | | Page 8, lines 4 – 7 |
| Introduction |  | |  |
| Background and rationale | 6a | | Pages 4 and 5 (lines 1 – 12) |
|  | 6b | | N/A |
| Objectives | 7 | | Page 5, lines 11 – 12 and page 5, lines 18 - 23 |
| Trial design | 8 | | Page 5, lines 18 – 25 and page 6, lines 1 - 2 |
| Methods: Participants, interventions, and outcomes | | | |
| Study setting | | 9 | Page 6, lines 5 - 7 |
| Eligibility criteria | | 10 | Page 6, lines 8 - 24 |
| Interventions | | 11a | Page 7, lines 1 - 12 |
|  |  | 11b | N/A |
|  |  | 11c | N/A |
|  |  | 11d | Page 7, lines 13 - 17 |
| Outcomes | | 12 | Page 7, lines 22 – 26 and page 8, lines 1 - 2 |
| Participant timeline | | 13 | Page 10, lines 18 - 20 |
| Sample size | | 14 | Page 8, lines 10 - 19 |
| Recruitment | | 15 | N/A |
| **Methods: Assignment of interventions (for controlled trials)** | | | |
| Allocation: | |  |  |
| Sequence generation | | 16a | Page 7, lines 1 - 3 |
| Allocation concealment mechanism | | 16b | N/A |
| Implementation | | 16c | N/A |
| Blinding (masking) | | 17a | Page 8, lines 4 – 7 |
|  | | 17b | N/A |
| **Methods: Data collection, management, and analysis** | | | |
| Data collection methods | | 18a | Page 8, lines 22 - 26 |
|  | | 18b | N/A |
| Data management | | 19 | Page 8, lines 11 - 15 |
| Statistical methods | | 20a | Page 8, lines 22 – 26 and page 9, lines 1 - 8 |
|  | | 20b | N/A |
|  | | 20c | N/A |
| **Methods: Monitoring** | | | |
| Data monitoring | | 21a | Endpoint Adjudication Committee and Data Monitoring Committee: page 8, lines 4 - 7 |
|  | | 21b | N/A |
| Harms | | 22 | N/A |
| Auditing | | 23 | N/A |
| Ethics and dissemination | | | |
| Research ethics approval | | 24 | Page 9, lines 11 - 23 |
| Protocol amendments | | 25 | N/A |
| Consent or assent | | 26a | Page 9, lines 14 - 19 |
|  | | 26b | N/A |
| Confidentiality | | 27 | Page 8, lines 22 - 25 |
| Declaration of interests | | 28 | Page 11, line 18 |
| Access to data | | 29 | Statement requested and present in the submission form |
| Ancillary and post-trial care | | 30 |  |
| Dissemination policy | | 31a |  |
|  | | 31b |  |
|  | | 31c |  |
| Appendices | |  |  |
| Informed consent materials | | 32 |  |
| Biological specimens | | 33 | N/A |
